# Homicide Using an Air Weapon

**DOI:** 10.5811/cpcem.2019.6.42982

**Published:** 2019-07-22

**Authors:** Benjamin Mogni, Sarah Maines

**Affiliations:** * Rush University Medical Center, Department of Emergency Medicine, Chicago, Illinois; † Kentucky State Medical Examiner’s Office, Frankfort, Kentucky

## Abstract

The debate over the lethality and ownership of modern, high-powered weapons has recently grabbed the headlines. High-velocity air weapons, advertised as starter guns for children, can cause lethal injuries despite non-lethal appearing wounds. Presented is a rare case of a modern, high-powered air weapon used in a homicide. A literature search yielded reports of only three previous murders by air weapon in the United States and only one involving injury to the thorax. In the current case, the killer used a diabolo pellet to penetrate the chest. The pathway tracked through the sternum, piercing the anterior pericardial sac and perforating the right ventricle, which led to a pericardial effusion. The pellet embolized to the left pulmonary artery and eventually the vasculature of the left lung. Cause of death was a penetrating gunshot wound of the chest most likely leading to cardiac tamponade. This case exemplifies several important characteristics of penetrating chest trauma from air guns: first, air rifles, with exit velocities up to 1200 feet per second, can kill and have been used in accidental deaths, homicides and suicides; secondly, diabolo pellets may embolize just as bullets can; and lastly, minor external damage may mask major internal destruction.

## INTRODUCTION

**Image 1:**
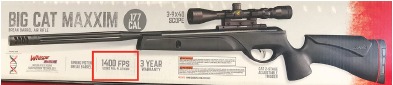
Example of an air rifle.

An air-powered gun is defined as a weapon that uses the expanding forces of compressed air or gas to propel a projectile.^1^ Retail stores advertise air weapons, shown in Image 1, as starter or recreational guns for children, but fail to warn citizens about the lethal threat they pose. While these injuries may appear non-lethal, they can cause deadly end organ damage. The literature on air weapon deaths yielded only three murders in the United States (U.S.), and only one of these described a gunshot wound to the chest. Case reports involving accidental deaths usually describe children with injuries to the head, neck and eyes.^1^–^8^ In reported cases involving penetration of the thorax, death resulted from laceration of the pulmonary artery, aorta or right ventricle.^9^–^11^ We discuss the homicide of a 31-year-old male killed by a penetrating injury of the right ventricle via air weapon, which led to pericardial tamponade. In addition, we review the literature.

## CASE REPORT

Emergency medical technicians arrived to find a man lying on the sidewalk for an unknown period of time. They provided Advanced Cardiac Life Support care, intubated the man, and placed him on a cardiac monitor. The reading showed asystole. He was transported to a local area hospital and pronounced dead after a resuscitation attempt. Medical staff noted a small, possible bullet wound over the patient’s sternum. The county coroner requested an autopsy.

On autopsy, the patient was a 68.5 inches (1.74 meters [m]) tall male weighing 174 pounds (lbs) (78.9 kilograms [kg]) with a penetrating gunshot wound of the midline chest, 0.19 inch (4.76 millimeters [mm]) in diameter and located 18.25 inches (46.4 centimeters [cm]) from the top of his head. Black cutaneous discoloration surrounded the wound and did not wipe away. From 11 o’clock to 6 o’clock the discoloration’s thickness was 0.13 inch (3.18 mm) wide, and from 6 o’clock to 11 o’clock it was 0.19 inch (1.59 mm).

**Image 2:**
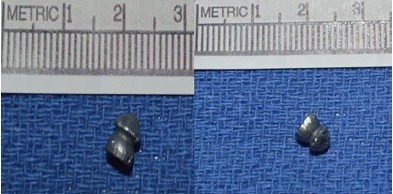
Diabolo pellet used in the homicide.

The forensic pathologist tracked the pathway of the projectile through the sternum; it pierced the anterior pericardial sac and penetrated the right ventricle of the heart, eventually entering the vasculature of the left lung. A diabolo air-rifle pellet, shown in Image 2 and described as hourglass or mushroom shaped with a hollow base, was recovered from the left pulmonary artery. The trajectory of the projectile was front to back, with minimal left to right or up-down deviation. Injuries outside of the heart included emphysema of the mediastinal soft tissue, hemorrhage of the mediastinal soft tissues and the left hilar soft tissues, and hemoaspiration. The patholgoist also noted a 500 milliliter (mL) hemopericardium and a 250 mL left hemothorax. Cause of death was a penetrating gunshot wound of the chest most likely leading to cardiac tamponade.

The police investigation revealed that a Game Big Cat 1200 air rifle had been used in the shooting. This gun can shoot at a top speed of 1200 feet per second (ft/s). It can only shoot a 0.18-inch (4.57 mm) air pellet.

## DISCUSSION

The danger of air weapons was evident as far back as the early 1800s when they were used in the Napoleonic wars.^4^ In their earliest days, air rifles held several advantages over powder firearms. These weapons were quieter, did not produce a flash or smoke, and a soldier could discharge 20 rounds in the same time it took him to reload a musket. However, the air weapon’s delicate nature did not hold up in combat.^11^ While today’s air rifles feature increased accuracy, automatic capability, and the power of modern firearms, they are no longer a weapon of war; rather they are advertised to children as a recreational device.^9^,^11^

In the late 1960s, the U.S. and the United Kingdom (UK) both adopted gun control laws. The U.S. excluded air weapons, including those using carbon dioxide, from being classified as firearms.^9^ (It is important to note that manufacturers did not introduce high-powered air rifles until the early 1970s.^6^,^12^) Currently, only 28 states have laws regulating air rifles; some states classify them as firearms while others exclude air rifles from this classification.^5^ In 1980, U.S. retailers sold over three million air weapons.^6^

**CPC-EM Capsule**

What do we already know about this clinical entity?

Ocular trauma is the most common presentation of air weapon injury in the emergency department. Most deaths from an air weapon injury are caused by intracranial penetration by the pellet.

What makes this presentation of disease reportable?

Penetration of the myocardium with an air rifle rarely occurs. Most deaths happen after the pellet enters the cranium. Furthermore, homicides are seldom committed with air weapons.

What is the major learning point?

Physicians should not underestimate the injury caused by an air weapon. A small external wound is often misleading. Furthermore, physicians should counsel patients on the dangers of these guns.

How might this improve emergency medicine practice?

This case report hopes to teach physicians to completely investigate air weapon wounds. Furthermore, we hope physicians will counsel patients on the dangers these weapons pose to children.

The Firearms (Dangerous Air Weapons) Rules of 1969 in the UK specified the maximum kinetic energy that an air pistol and air rifle could eject their projectile as follows: at six feet per pound (ft/lb) (8.1 joules [J]) for an air pistol and 12 ft/lb (16.3 J) for an air rifle. Any greater energy categorized the gun as a firearm and required a certificate for possession. Use of carbon dioxide classified the air weapon as a firearm.^4^ In Scotland, the total number of injuries due to air weapons decreased from 2377 in 2002/2003 to 299 in 2014/2015. Of these reported injuries, authorities reported zero to three fatalities per year. In 2017, Scottish authorities began requiring licenses for possession of an air weapon.^13^ No data has been published yet on whether this requirement has further decreased air weapon-induced injuries or deaths.

Physicians have long been aware of the ophthalmologic dangers of air weapons.^7^,^14^–^22^ Bowen in 1973 showed that out of 105 cases of eye injury due to air weapon, 43% of children had significant residual visual deficits and 20% of children with a globe rupture from an air weapon required removal of the eye.^15^ However, these weapons also cause injuries outside of the eye and even death.

The greatest factor in any death by projectile is the kinetic energy of the projectile. The equation for kinetic energy is KE = ½mv^2. Therefore, impact velocity of the pellet claims the largest component involved in pellet gun deaths.^23^ The farther the shooter stands from the victim, the lower the impact velocity and the lower the kinetic energy. Diabolo pellets consist of light material and their speed deteriorates quickly. Therefore, the pellets’ exit velocity, distance traveled, and which tissue they hit determine the damage done.^11^,^24^ In the early 1960s, the U.S. Army determined that a projectile requires 58 ft/lb (78.6 J) of force to create a casualty.^16^ With a force between one and 10.9 ft/lb (1.4 J and 14.8 J), air weapons fail to satisfy this criterion; however, they could cause injury or death if the pellets were to strike certain anatomical locations.^1^

The most recent data from 2000 showed 21,840 injuries from air weapons in the U.S. Unfortunately, children prove most vulnerable to injury and death from air rifles, especially when unsupervised.^24^,^25^ In 1995, the Centers for Disease Control and Prevention (CDC) reported air weapon injuries over a two-year time span. They reported that children and teenagers, 18 and younger, suffered 81% of the injuries.^15^,^16^ Percentages on anatomic location of injury vary by series. However, the CDC report stated that 6% occurred to the eye, 25% to the head and neck not including the eye, 54% to the extremities, and 15% to the trunk.^16^ In 1997, Scribano reported that minor injuries comprised 74% of injuries related to air weapons presenting to the emergency department (ED).^18^

The ammunition used in these weapons varies depending on the gun. The classic Daisy BB Gun will fire a pellet 0.18 inch (4.57 mm) in diameter and weighing 0.013 ounce (0.36 grams [g]). An air rifle will fire a diabolo pellet, a mushroom-shaped, gray projectile with a hollow bottom shown in Image 2. Air rifles typically fire two calibers of diabolo pellets: a 0.18-inch (4.57 mm), 0.53-g (0.019 ounce) pellet, or a 0.22-inch (5.59 mm), 0.97-g (0.034 ounce) pellet. These weapons can use several mechanisms to fire the pellets. 1

Air weapons use three main propulsion arrangements to propel their pellets: spring-piston, pneumatic, and carbon dioxide. In a spring-piston weapon, a spring moves the piston and compresses air in a chamber. When released, the spring expels the air from the chamber and the pellet exits the barrel. The pellet travels approximately 1000 ft per second (/s) (305 m/s).^1^ A pneumatic air gun employs a system of pumps, with more pumps imparting a greater velocity, to deploy the projectile up to 770 ft/s (235 m/s).^26^ A carbon dioxide-powered gun exploits compressed carbon dioxide. This scheme maintains approximately the same speed as a spring-piston air gun but varies with temperature.^1^

**Image 3:**
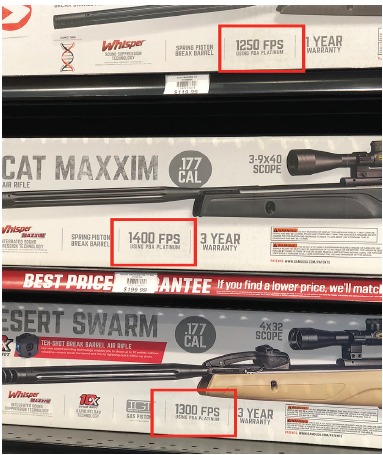
Example of maximum velocity.

A process known as “dieseling” increases the velocities of all these weapons. Dieseling occurs when regular greasing of the barrel leaves excess oil, which the pellet ignites as it hurtles through the barrel.^4^,^27^ Older versions of these guns, such as the classic Daisy BB Gun, discharged a projectile between 275 ft/s and 350 ft/s (84 m/s to 107 m/s). The literature reports that newer versions can fire a projectile as fast as 1200 ft/s (366 m/s) but most reach a velocity of 900 ft/s (274 m/s).^1^,^11^ A trip to a department store by this author found several air weapons that could fire 1300 ft/s and 1400 ft/s as shown in Image 3. This surpasses the velocity of several, low-power handguns including a 9-mm, which Dimaio reported as 1140 ft/s (347 m/s).^1^,^5^,^6^,^9^,^11^ With a velocity this high, these bullets may cause damage to any part of the body.

By firing pellets at pig eyes, the authors of one study determined that corneal perforation and globe rupture occurs 50% of the time at a velocity of 246 ft/s to 249 ft/s (75 m/s to 76 m/s) using a 0.18-inch (4.50 mm), 0.013 ounce (0.36 g) BB.^1^,^17^ Dimaio established skin penetration of human lower extremities results at 331 ft/s (101 m/s) for a 0.18-inch (4.57 mm), 0.01-ounce (0.54-g) pellet and 245 ft/s (75 m/s) for a 0.038-ounce (1.07 g), 0.22-inch (5.59 mm) pellet.^28^ Other reports place bone penetration around 350 ft/s (107 m/s).^1^ However, the damage depends on the kinetic energy and, therefore, the weight as well. The lighter the projectile, the greater the velocity needed for penetration. Skin thickness and subcutaneous tissue will also affect the degree of penetration. Children tend to have thinner skin than adults and, therefore, a lower velocity will be needed to break the skin.^1^

Smedra-Kazmirska et al. studied the velocity air pellets maintain for a certain distance and their force on impact. The highest velocity came from the “BMK 19”-0.18 inch (4.5 mm) air rifle with a velocity of 886 ft/s (270 m/s) and carrying an initial kinetic energy of 12.19 ft-lb (16.58 J). Depending on pellet type used, this led to penetration of a pellet into ballistics gelatin between 1.97 inches and 3.7 inches (50 mm and 94 mm) when fired from 65.6 ft (20 m). The study showed that several air rifle models available in 2013, including the “BMK 19”-0.18 inch (4.5 mm), could penetrate the pericardium from a distance of 65.6 ft (20 m).^29^

From 1982 to 1996, 33 deaths from air weapons occurred in the U.S.^6^ From 1990 to 2000, American authors reported 39 deaths involving air weapons, 32 of which were children younger than 15 years old.^12^ Cranial damage resulted in death most often. The pellet typically entered transnasally, transocularly or transtemporally, where the bone is the thinnest.^11^,^30^,^31^ Several authors warn that children with these penetrating head injuries appear fine and maintain consciousness but may rapidly deteriorate. 25,32 However, injuries to the abdomen and thorax may result in fatality as well.

Penetrating wounds to the chest and abdomen carry significant morbidity and mortality. Chest injuries classically involve damage of the myocardium, aorta, or pulmonary vessels leading to cardiac tamponade or hemothorax.^1^,^4^–^6^,^9^–^11^,^18^,^24^,^33^–^36^ While perforating injuries from air weapons to the abdomen commonly result in visceral injury, they can also injure the abdominal aorta.^5^,^6^,^18^,^37^,^38^ Furthermore, physicians often underestimate the injury potential of air weapon gunshot wounds to the chest and abdomen.^37^ Physicians must understand that air-weapon pellets carry a propensity to embolize once in the bloodstream.^34^,^39^ (The old fear of lead poisoning from a retained air-weapon pellet appears obsolete, as the modern alloy composition of the ammunition does not elevate serum lead levels.^19^) Accidental trauma, suicide, or homicide may precipitate these injuries and deaths.

Although uncommon, suicide attempts typically involve penetrating injuries to the head, but also may involve the abdomen and chest.^30^,^33^,^40^–^42^ The CDC reported in 1995 that 0.1% of ED visits for air-weapon injuries were from suicide attempts.^16^ Campbell-Hewson et al. presented a series of nine attempted suicides by air weapon and three completed suicides. The authors proposed that the availability of high-power firearms in the U.S. precludes air weapons as common weapons for suicide and murder.^41^

In the U.S., only three authors reported homicides with an air rifle since 1975. Dimaio described the first murder by air rifle portraying two teenage boys involved in a heated argument. 43 The younger of the two grabbed an air rifle and fired from only a few feet away, striking the older boy in the medial half of the right upper eyelid. The pellet travelled through the soft tissue of the orbit superiorly to the globe and entered the cranial cavity. The pellet caused damage to both hemispheres of the brain. The 17-year-old only survived for an hour and a half following the shot. Green in 1980 reported a case in which a gunshot wound to the head killed a man.^44^ It appears two pellets were loaded into the chamber in a technique called “piggybacking.” Piggybacking occurs when two projectiles are loaded in the chamber. When fired, the increased mass augments the momentum of the pellets and thus intensifies the impact energy.^4^,^6^

In 2010, Bligh-Glover reported a drive-by shooting where an air-rifle bullet struck the victim’s left chest. The projectile perforated the pulmonary vasculature leading to a hemothorax. The bullet further struck the left ventricle but did not penetrate the myocardium. In 2001 in Japan, Ng’walali reported that a 0.03-ounce (0.9 g) and 0.22-inch (5.59 mm) diameter pellet perforated an elderly woman’s myocardium leading to cardiac tamponade and death. The pellet embolized to the left subclavian artery. 35

## CONCLUSION

Most emergency physicians (EP) do not observe homicides or any other manner of death caused by air weapons because these events are rare. This case portrays only the fourth homicide using an air weapon that has been reported in the U S. Although decreasing in popularity, air weapons still pose a threat that physicians should be prepared to treat and counsel patients about. Adults must supervise children using air weapons. Furthermore, any weapon that can penetrate the pericardium from 20 m away should not be viewed as a toy. When these injuries present to the ED, the EP should treat them as penetrating trauma.^9^ Likewise, an EP should not underestimate an air-weapon injury, as the outside trauma may appear minimal despite extensive internal damage.^18^,^37^ Without any soot around the wound the EP cannot distinguish between a wound inflicted from a short distance or long range.^16^

Several cultural factors enhance the dangers of these weapons. The physician must consider these aspects when counseling. First, many people view air guns as toys. Their low cost reinforces this perception. Also, lack of regulations and restrictions allow their simple acquisition in stores or online.^16^,^37^,^40^ Our online search found dozens of air rifles on sale for as little as $70.

Felons execute homicides with these guns; and in their lowest moments people commit suicide with these weapons. Physicians have debated the need for this legislation in the past with no clear consensus.^8^,^20^ Perhaps it is time to revisit the topic, as gun-control legislation specifically addressing air weapons would increase public awareness of the danger.
